# Enrichment strategies to enhance genome editing

**DOI:** 10.1186/s12929-023-00943-1

**Published:** 2023-07-01

**Authors:** Nanna S. Mikkelsen, Rasmus O. Bak

**Affiliations:** Department of Biomedicine, Aarhus University, Høegh-Guldbergsgade 10, Bldg. 1115, 8000 Aarhus C., Denmark

**Keywords:** CRISPR/Cas, Enrichment, Selection, Reporter, Integration, INDELs, HDR, NHEJ, FACS, MACS, Surrogate reporter, Genetically modified cells, Genome editing, Co-editing

## Abstract

Genome editing technologies hold great promise for numerous applications including the understanding of cellular and disease mechanisms and the development of gene and cellular therapies. Achieving high editing frequencies is critical to these research areas and to achieve the overall goal of being able to manipulate any target with any desired genetic outcome. However, gene editing technologies sometimes suffer from low editing efficiencies due to several challenges. This is often the case for emerging gene editing technologies, which require assistance for translation into broader applications. Enrichment strategies can support this goal by selecting gene edited cells from non-edited cells. In this review, we elucidate the different enrichment strategies, their many applications in non-clinical and clinical settings, and the remaining need for novel strategies to further improve genome research and gene and cellular therapy studies.

## Introduction

The development and widespread use of gene editing technologies such as zinc finger nucleases (ZFNs), transcription activator-like effector nucleases (TALENs), Meganucleases, and clustered regularly interspaced short palindromic repeats (CRISPR)/CRISPR-associated (Cas) systems and derivatives hereof have provided great opportunities for site-specific genome editing [1, 2]. Especially the simplicity of the CRISPR/Cas system has made it the preferred choice for genome editing. The RNA-guided type II CRISPR/Cas system consists of a Cas9 nuclease, which is guided to a specific target site by a chimeric single guide RNA (sgRNA) [1]. Site-specific genome editing is achieved by introducing a nuclease-induced double-strand break (DSB) to the DNA and relying on the endogenous cellular repair mechanisms to alter the genome [3]. The most prevalent repair mechanism is the error-prone non-homologous end joining (NHEJ), which is active throughout the cell cycle by direct ligation of DNA ends, often resulting in small insertions or deletions (INDELs) at the site of the break due to end-processing during repair [4]. If a DSB lies between two homologous repeat sequences in the same direction, the single-strand annealing (SSA) pathway can anneal these sequences and mediate deletion of one repeat and the intervening sequence to seal the DSB [5]. In contrast, homology-directed repair (HDR) uses an exogenous DNA template for targeted integration of transgenes facilitated by homology arms identical to the DSB flanking sequence [6].

## The bottlenecks of genome editing

Despite the great potential of gene editing technologies, not all exhibit high activity and efficiency. Gene editing efficiency can vary widely between genomic target loci and among cell types. Particularly, integration of transgenes into the genome relying on HDR-mediated integration (“knock in”) is an inefficient process suffering from low editing rates compared to gene disruptions mediated by NHEJ (“knock out”). HDR-mediated site-specific integration can in some scenarios be achieved with high efficiency in cell types and loci particularly permissive to gene editing [7], but is generally restricted to < 30% of cells obtaining a targeted integration in most cases [8–10]. Furthermore, HDR activity is largely restricted to the S and G2 mitotic phases of the cell cycle, where homologous sister chromatids are present for natural DNA repair. Therefore, HDR can display limited efficiency in quiescent or slowly cycling cells [11].

Depending on the desired edit and target locus, numerous factors and challenges can impair genome modification efficiencies, some of which include: (1) inefficient delivery of the gene editing system [12, 13], (2) toxicity caused by delivery modality and exposure to gene editing components [14–16], (3) restriction to a narrow target sequence window with inefficient nuclease target sites, which could be caused by complex target sequences with repetitive elements, unusual GC content, or a dense chromatin state [17, 18], and (4) quiescent cells without an active endogenous repair machinery [11, 19]. Consequently, suboptimal gene editing efficiencies may hamper the use of gene editing technologies for some applications [1, 20].

More recent genome editing technologies like base editing [21, 22] and prime editing [23] do not rely on the formation of a DSB, and are thus not restricted by inactive endogenous repair machinery. However, other challenges for these technologies persist. Base editing has recently shown great editing efficiencies and a potential for treating various monogenic diseases [24, 25]. Unfortunately, base editing is limited to point mutations only, and the purity of the editing outcome with base editors is a concern when there is more than one target base in the editing window, thereby limiting the potential of base editors [26, 27]. Reducing the size of the editing window with newer base editors to increase specificity might in turn reduce editing efficiency and limit the genomic sites that can be targeted because of PAM constraints. Instead, enrichment of base edited cells may be an alternative to improve editing efficiencies.

Prime editing exhibits a high rate of precise editing to unwanted INDEL formation [28]. However, prime editing efficiencies can be very low, suffer from impure edits, is restricted to small edits, and often requires extensive optimization [20, 28, 29]. Any approach that can substantially increase editing outcomes is critical for its forthcoming use.

Other gene editing technologies for the integration of larger DNA sequences into the genome without reliance on endogenous repair pathways have emerged. These include CRISPR-associated transposon (CAST) systems, which are transposons that have co-opted Cas proteins for precise RNA-guided DNA insertion. CAST-mediated editing has been demonstrated in prokaryotes [30–33] and recently in mammalian cells albeit with very low integration efficiencies (< 0.1%) [34], which can be increased with enrichment [35]. Similarly, Cas nucleases combined with site-specific recombinases have enabled integration of larger DNA segments into human cellular genomes. The platform termed Programmable Addition via Site-specific Targeting Elements (PASTE) facilitated multiplexed insertions of large DNA cargo at multiple genomic loci in both human cell lines, primary human T cells, and non-dividing primary human hepatocytes with high precision. [36] Another approach based on a twin prime editing strategy (TwinPE) yielded 9% efficiency of correcting a large sequence inversion associated with Hunter syndrome in human cells [37].

**Fig. 1:**
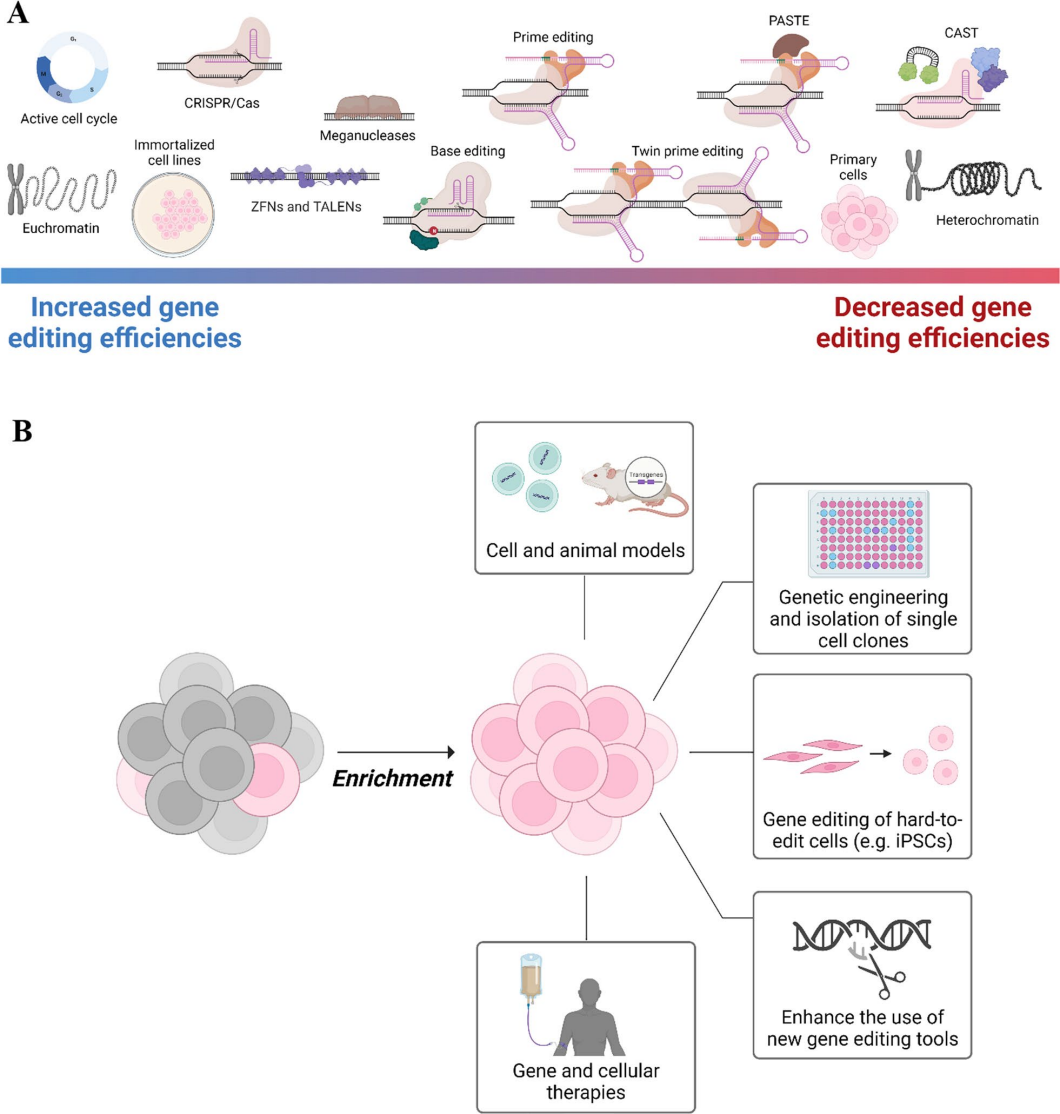
Enrichment rationale and applications. **A** Different conditions and choice of gene editing technology influence gene editing efficiencies. These are ordered based on their approximate impact on genome editing efficiency, but can be subject to high variability. **B** Applications for enrichment of gene edited cells include engineering of cell and animal models, engineering and isolation of single cell clones, editing of hard-to-edit cell types, making new gene editing tools more applicable, and facilitating the use of gene editing in gene and cellular therapies

Despite great potential of these novel DSB-free gene editing technologies, further improvements are required to generalize these genome editing modalities and achieve robust editing across all desired target sites. Figure 1A summarizes some of the bottlenecks affecting genome editing efficiencies including cell type, cell and gene state, and the selected genome editing tool.

## Improving gene editing efficiencies

Various strategies have been developed to improve the yield and accuracy of correctly edited cells. These include approaches to improve activity and/or specificity by engineered Cas enzymes [38–44], sgRNA design optimizations [45, 46], improved design and delivery of the nuclease and HDR templates [7, 47, 48], manipulation of repair pathways to adjust NHEJ:HDR ratio [49, 50], cell cycle control [19, 51–53], retargeting undesired INDELs by recursive editing for a subsequent opportunity to perform the desired HDR-mediated integration [54], introducing non-cleavable Cas9 target sequences (CTSs) in the HDR template to facilitate Cas9-mediated nuclear import of the template [55], or recruitment of the HDR template to the target site by direct fusion to the nuclease [56]. Several strategies have been investigated, which are reviewed extensively elsewhere [20].

## Selection of gene edited cells

A different overall strategy to improve the frequency of gene edited cells in a population aims to select edited cells from unedited cells. There are various means to do this based on negative or positive selection and using physical or biological separation methods. Some approaches may enable close to 100% selection efficiency while others merely enable a small enrichment. Enrichment may find various uses within basic biological studies as well as in clinical applications. CRISPR/Cas has democratized the generation of genetically engineered cell lines for studies of genotype–phenotype relationships, but generating a clonal cell line with the desired genotype may require labor-intensive screening of hundreds of clones to identify a correct one. Enrichment may facilitate the direct identification and isolation of very infrequent genotypes in a population or at least vastly increase the likelihood of identifying a correct clone, thereby reducing the labor intensity [57, 58].

Enrichment strategies can also prove valuable in a clinical setting. For some applications, large inter-patient variability in gene editing efficiency can be a limiting factor. Enrichment of correctly gene edited cells for therapies could reduce this issue and potentially assure a product for all patients. In other cases, gene editing efficiencies are not high enough to provide a therapeutic effect. One example is hematopoietic stem cell (HSC) therapies where edited and unedited cells compete during engraftment and hematopoiesis. For some therapies like x-linked severe combined immunodeficiency (X-SCID), there is a large survival benefit during lymphopoiesis for correctly edited cells, which means that a very small fraction of edited HSC is believed to suffice to provide a therapeutic benefit. For other hematopoietic disorders like chronic granulomatous disease (CGD), such enhanced survival advantage does not exist, and low editing rates would not provide a therapeutic effect [59].

New treatment modalities for Cystic Fibrosis also investigate genome editing strategies. Previous studies have shown that the presence of 10–25% *CFTR*-expressing cells is sufficient to restore *CFTR* function [60, 61]. Implementing enrichment of CRISPR/Cas edited cells increased the frequency of *CFTR* edited cells from 15 to 80%, thereby greatly exceeding the desired minimum editing level [60].

Cellular immunotherapies have revolutionized treatment of especially difficult-to-treat CD19 + hematological malignancies, and anti-CD19 Chimeric Antigen Receptor (CAR) T cell therapies are commercially available from several companies. Here, the patient’s own T cells are engineered to express the CAR using lentiviral vectors, and notably, the inter-trial and inter-patient variabilities are significant regarding CAR expressing cells. Different clinical trials have reported 5 to > 90% of T cells expressing the CAR following manufacturing [62–66], and with a desired release criteria of > 10–20% CAR T cells, improvements are necessary [67, 68]. This might prove particularly important when using CRISPR/Cas approaches to insert the CAR gene as efficiencies may be inferior to lentiviral delivery. The possibility to enrich engineered CAR T cells before infusion into patients might generate more successful CAR T cell therapies and potentially facilitate the transition into allogeneic cell products [69]. Furthermore, for such kind of cell therapy with in vitro expansion of the engineered cells, there might be a benefit in manufacturing costs since undesired therapeutically irrelevant cells are not included during the expansion process.

Strategies to enrich gene edited cells would facilitate the further use of programmable nucleases for many applications including engineering of cell and animal models, engineering and isolation of single cell clones, engineering hard-to-edit cell types, making new gene editing tools more applicable, and facilitate the use of gene editing in gene and cellular therapies (Fig. 1B). In this review we describe different enrichment strategies developed for the selection and isolation of gene modified cells from unmodified cells.

## Enrichment of transfection- or transduction-positive cells

**Fig. 2:**
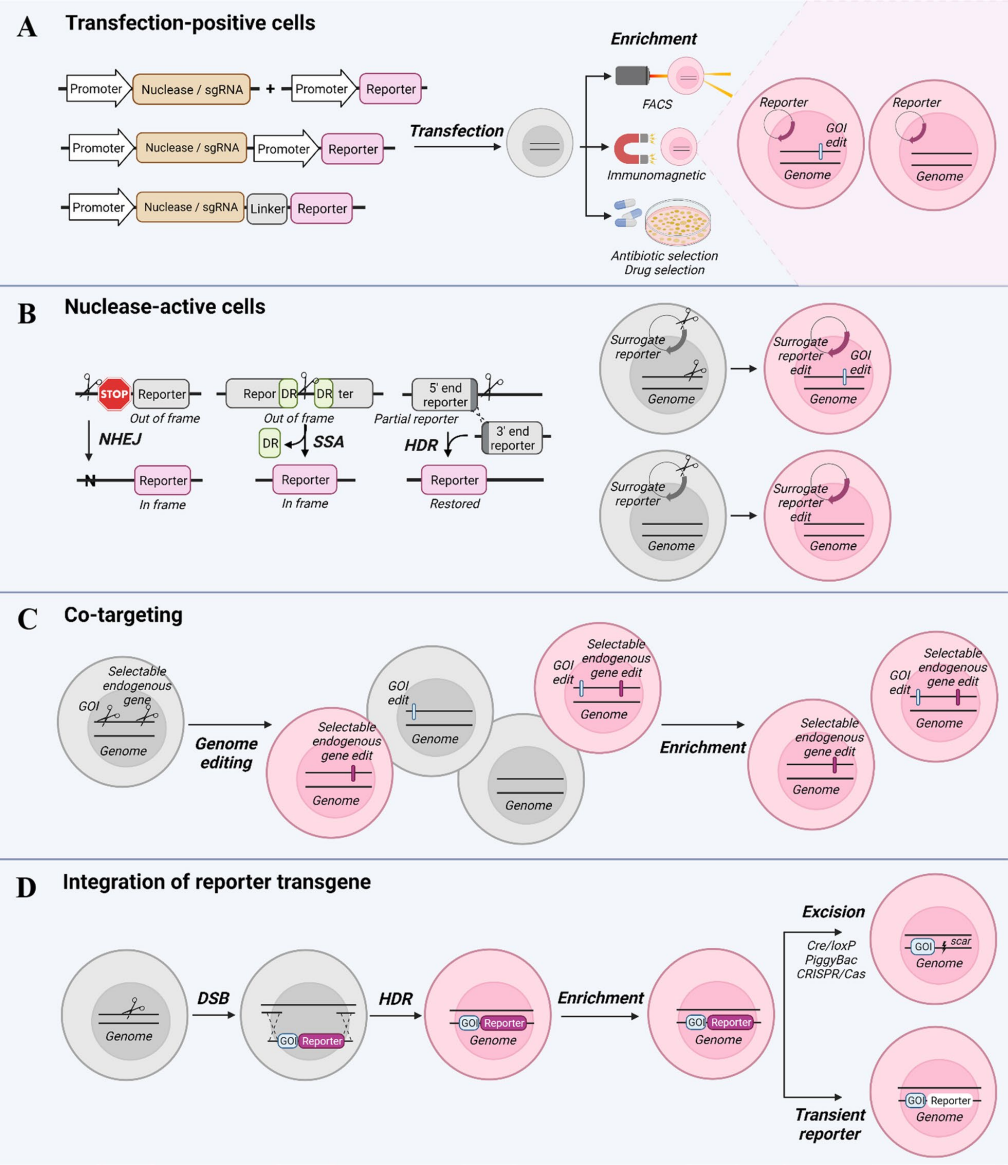
Different enrichment strategies. **A** Enrichment of transfection-positive cells with selectable reporter genes. **B** Enrichment of nuclease-active cells using surrogate reporters based on NHEJ-, SSA-, or HDR-mediated restoration of a reporter gene. **C** Enrichment by co-targeting. **D** Co-integration of a selectable reporter gene to enrich for HDR-mediated transgene integration events. The reporter gene can subsequently stay permanently in the genome, be excised, or become silent due to induced, transient reporter expression. Constructs are not to scale. GOI (gene of interest)

The first hurdle to overcome for genome editing is the delivery of the genome editing system into cells. A simple obvious approach is to enrich for cells that have efficiently taken up the genome editing system. This can be done for example through linkage with a selectable reporter such as fluorescent, antibiotic, or antigenic reporters, which allows for selection of cells efficiently transfected with the genome editing system. Introducing a reporter can be achieved by simple co-transfection of the reporter only allowing for enrichment of transfection-competent cells or by coupling the selectable reporter to the sgRNA or the nuclease for example via a self-cleaving 2A sequence (T2A) allowing for enrichment of nuclease- or sgRNA- expressing cells (Fig. 2A) [70–81].

This strategy proved especially valuable for multiplexed gene editing in primary CD34 + HSPCs. Here lentiviral sgRNA vectors containing fluorescent markers allowed for traceable and selectable multiplexed editing. Robust double knock outs of cell surface molecules CD45 and CD44 with an efficiency of ~ 70% was achieved in HSPCs. Multiplexed knockouts were also demonstrated for *STAG1* and *STAG2* genes as well as for the *AHR* and *LSD1* or *RCOR1* genes resulting in marked CD34+ HSPC expansion [82].

## Enrichment of nuclease-active cells using exogenous surrogate reporters

Another way of not only selecting for transfected cells, but also nuclease-active cells involves the use of surrogate reporters that report on NHEJ [83–88], SSA [84, 85, 89–92], or HDR activity [83, 84, 90, 92, 93]. The use of surrogate reporter systems to enrich for genome edited cells is based on the premise that engineered nucleases able to edit the co-delivered surrogate reporter have a higher probability of also editing the genomic target site, since cells proficient for genome editing at one locus are more likely to be proficient for editing at another locus, called co-targeting or co-selection [94, 95]. These surrogate reporter systems show improved enrichment of gene edited cells compared to simple selection of transfection-positive cells [96, 97]. Various exogenous surrogate reporters based on NHEJ-, SSA-, or HDR-mediated editing by engineered nucleases have been developed for the enrichment of genome editing events. An overview of the three surrogate reporter types are presented in Fig. 2B.

### NHEJ-based exogenous surrogate reporters

NHEJ-based exogenous surrogate reporters for enrichment of gene edited cells are based on a nuclease target sequence inserted to shift the reading frame of a selectable reporter gene. Only new INDEL events can restore the reading frame and the expression of the selectable reporter. Kim et al. were the first to develop an NHEJ-based surrogate reporter system with a frameshifted GFP reporter to enrich for gene edited cells demonstrating up to 39-fold enrichment of INDELs (from 0.62 to 24%) in the *TP53* gene in the sorted GFP + cell population versus the unsorted population [98]. Enrichment of gene edited cells was also achieved using similar surrogate reporter systems relying on enrichment by magnetic selection of a truncated mouse MHC class I molecule (H2-kk) antigen or antibiotic selection using a hygromycin resistance gene (hygroR) as selectable frame-shifted genes [99]. Even though high numbers of reporter-positive cells can be observed with this strategy, perhaps due to excess reporter plasmids in the cells [97], NHEJ-mediated INDEL formation occurs with an uncontrollable outcome, which means that on average only 1/3 of the surrogate reporters would generate an in-frame selection gene. Additional changes have been implemented to increase the sensitivity of the reporter to 2/3 by including two different out-of-frame reporter genes (3n + 1 and 3n + 2). The new NHEJ-based surrogate reporter system was also developed to enrich for CRISPR/Cas-mediated genome editing events by both FACS, immunomagnetic selection, and antibiotic selection. Up to 11-fold enrichment (from 2.8 to 31%) of nuclease-induced mutations at the target site was observed compared to the unselected cell population [96]. Similar enrichment efficiencies have been achieved in other studies using this and similar surrogate reporters. [100–103].

Despite the ease of using surrogate reporters, a new reporter needs to be cloned for each nuclease target site to be edited, since the most efficient enrichment is achieved if the nuclease targets the same sequence as the genomic sequence. To solve this issue, NHEJ-based surrogate reporters have been developed with the target sequence flanked by restriction sites allowing for easy exchange of the target sequence [97]. More advanced approaches have also been developed that include 17 target sites in a row generating a single reporter able to enrich for edits at any of the 17 target sites [104]. However, this strategy increases the challenge of arranging all target sites so that a premature stop codon does not occur in the reporter, which would otherwise compromise the functionality of the reporter as it is required to be able to be turned on upon the right frameshifting INDELs. Nonetheless, this surrogate reporter achieved enrichment so 80% of reporter-positive cells contained the desired edit compared to < 10% in the reporter negative population [104].

### SSA-based exogenous surrogate reporters

Unlike the NHEJ-based surrogate reporter systems, avoidance of in-frame premature stop codons at the target sequence is not required for the SSA-based surrogate reporter system, thus simplifying its in silico design and broadening its applications. Instead, the selection reporter is disrupted by a nuclease target site flanked by direct repeats (DRs), which allows for restoration of the reporter upon SSA-mediated intramolecular repair of the DSB introduced at the nuclease target site by deleting one of the DRs along the region in between. Several SSA-based surrogate reporter systems have been described using both fluorescence [91, 103] and antibiotic resistance [103, 105], achieving increased INDEL rates from 8.7 to 97.9% in the reporter positive population [91].

A dual surrogate reporter system containing two different reporter cassettes was designed to also act as repair template for HDR, thereby potentially allowing enrichment of both INDELs and integration events. One reporter cassette can function as surrogate reporter for nuclease-activity and enrichment and the second reporter for knock-in and screening of biallelically targeted cells based on dual antibiotic selection yielding 6.7-fold enrichment (from 2.70 to 18.18%) of biallelic integrations compared to the use of only one reporter [8]. Comparison of NHEJ- and SSA-based surrogate reporter systems in one study revealed superior enrichment when utilizing an SSA-based surrogate reporter system achieving up to 34.8-fold enrichment (from 2.1 to 72.7%) of INDELs compared to non-selected cells with an optimal DR length of 200 bp [103]. However, one study has demonstrated the opposite, that the NHEJ-based reporter is most efficient [106].

### HDR-based exogenous surrogate reporters

HDR-based exogenous surrogate reporters enrich for cells that have both nuclease activity and an active HDR machinery, thereby potentially enriching for integration events. Different HDR-based surrogate reporter systems have been described, but all rely on restoration of a reporter gene by HDR-mediated repair of a DSB [107–110]. One system contains a truncated N-terminal part of a reporter gene (puromycin resistance (PuroR) or eGFP) followed by the nuclease target site and next a full length reporter gene with a stop codon instead of a start codon. Upon nuclease-induced DSB formation at the nuclease target site, the full-length reporter can be repaired by recombination with the N-terminal homologous part, thereby replacing the stop codon with a start codon and mediating reporter expression [107]. Similarly, disrupting the reporter by a nuclease expression cassette flanked by nuclease target sites mediated restoration of the reporter gene and simultaneous self-inactivation upon removal of the nuclease expression cassette to restore the reporter gene by HDR [108]. Another system restores a truncated puromycin resistance gene by intra-molecular HDR using a “universal” sgRNA target site not present in the human genome for introducing the DSB in the surrogate reporter. This identifies cells with HDR activity and a potential simultaneous integration in the genome if another sgRNA and matching HDR template was supplied. HDR-mediated editing of precise point mutations was increased up to 20.7-fold (from 2.22 to 45.93%) and HDR-mediated integration of an eGFP gene was enriched up to 8.9-fold (from 1.34 to 11.93%) with only 50 bp homology arms [109].

Most surrogate reporters enrich for both transfection-positive cells and nuclease-active cells if an additional reporter is included for assessing transfection efficiency (dual surrogate reporters). NHEJ and SSA-based reporters are best at enriching for knock out events, but all reporters can also be used to enrich for HDR events, since they enrich for nuclease activity [97]. However, these strategies are not able to directly enrich for specific editing events since they are only able to enrich for nuclease activity.

The biggest advantage of these episomal surrogate reporters is that they are transiently transfected and do not intentionally alter the genome, thereby constituting a scarless enrichment strategy. On the other hand, relying on plasmid surrogate reporters can potentially be a limitation since random integration of plasmid DNA into the genome can occur [111]. Furthermore, the introduction of a DSB both in the episomal reporter and in a genomic locus of interest, increases the risk of interference between the two sites and integration of the episomal reporter into the genomic locus. Absence of such unintended outcomes related to this enrichment strategy should be verified especially for clinically relevant applications. Another potential issue with surrogate reporters is an overestimation of editing efficiencies if relying on reporter expression for example to screen sgRNA efficiencies due to differences in chromatin state at the genomic loci versus the targeting site in the reporter [112].

## Enrichment of nuclease-active cells using endogenous reporters

The premise of co-targeting is also the foundation of another group of enrichment strategies. Contrary to the use of exogenous surrogate reporters, these strategies rely on inconsequential mutations made to endogenous genes to create a selectable phenotype (for example drug resistance). Hence, enrichment of cells is facilitated through a modification at a second unrelated endogenous locus (Fig. 2C). Avoiding the use of exogenous surrogate reporters makes this category of enrichment strategies more compatible with therapeutic applications, depending on the selection edit made to the genome, since no exogenous reporter DNA is introduced into the cells [113]. This co-targeting strategy was originally described in C. elegans [114, 115] and later applied to mammalian cells as well.

Moriarity et al. used this strategy to co-target the *HPRT* gene in CD34+ HSPCs. Cells lacking endogenous *HPRT* expression become resistant to the cytotoxic drug 6-thioguanine (6-TG) thereby allowing for enrichment of cells with a simultaneous knock out of either the *CCR5* or *ARTEMIS* gene by NHEJ. Up to 64.1% of the 6-TG resistant HSPCs presented were co-edited at the desired target site. [116] Another study applied the same co-targeting strategy and achieved enrichment of NHEJ-mediated *AAVS1* editing events to over 80% with co-targeting [117]. However, since *HPRT* is X-linked, 6-TG resistant cells arise from modification of only a single active allele, which may not provide an adequate selection pressure if biallelic co-targeting knockouts are desired [116, 118].Another endogenous co-targeting strategy for enrichment of NHEJ or HDR modifications at a second locus utilizes mutations in the Na+ /K+ ATPase gene *ATP1A1* that renders cells resistant to the inhibitor Ouabain (a cardiac glycoside). The authors achieved successful enrichment for CRISPR-induced INDELs and HDR events in both cell lines and in CD34+ HSPCs [113]. This strategy was further developed for induced pluripotent stem cells (iPSCs). Here they improved INDEL rates, whereas HDR rates were improved only at some loci, which could be attributed to differences in chromatin accessibility [119].

Other selectable targets for co-targeted enrichment of human primary cells include disruption of the *SLC35F2* gene making hPSCs insensitive to the anti-cancer drug YM155 [100], disruption of the *HBEGF* gene encoding the receptor for Diphtheria Toxin (DT) making iPSCs and primary human T cells insensitive to DT, which improved INDEL formation up to 14-fold (from ~ 5 to ~ 70%) and HDR-mediated integration at a second locus more than 35-fold (from ~ 0.2 to 6%) [120], disruption of the *B2M* gene followed by negative selection by for example FACS [118], or disruption of a pre-introduced temperature-sensitive (ts) mutation in the essential *TAF1* gene in cell lines rendering only edited cells temperature-resistant [121].

In a few cases a co-targeting-based enrichment strategy has been based on integration of a selection cassette at one genomic locus by HDR, thereby also enriching for HDR-mediated integration of another repair template at another independent locus. This has achieved up to 50-fold increased integration frequency at a second locus [122, 123].

These endogenous co-targeting strategies are in most cases more therapeutically applicable since no exogenous material is required to be introduced to the target cell population and the strategy might thereby be more suitable for use in primary cells. However, introducing two simultaneous DSBs to the genome can cause chromosomal translocations which is a primary driver of many cancers [124, 125]. Additionally, modifying an endogenous gene to create a selectable phenotype can also be problematic in some cases if it alters an essential or required gene function. Assuring that all desired target cells express the endogenous selection gene and checking for random escape from the selectable mutation should also be considered to confirm the enrichment potential of the strategy.

## Enrichment following reporter integration

A straightforward strategy to directly enrich for a specific genomic modification is to introduce a selectable reporter gene into the target locus possibly along a desired gene or cDNA to be integrated. This strategy has been applied in different variations. An overview can be seen in Fig. 2D.

### Reporter integration with permanent expression

Several studies have integrated reporter genes at specific genomic loci to track genome modification outcomes or to track endogenous genes and protein activity, localization, and dynamics [126–134]. This approach can be used for targeted disruption of a gene by integration of a reporter gene into the open reading frame of the target gene. The reporter gene enables direct tracking of cells with the disrupted target gene. It also enables enrichment for the integration of a target gene’s cDNA by utilizing a reporter tagged to this cDNA. Some reporter genes derived from endogenous genes can be clinically relevant since no foreign protein is introduced into cells. Especially, truncated signaling-inert membrane proteins, including *tNGFR* (truncated nerve growth factor receptor) and *tEGFR* (truncated epidermal growth factor receptor), have been used as reporters in clinical trials and shown to be safe [135–138]. However, constitutive overexpression and enrichment of a reporter like tEGFR in HSPCs would preclude patients from receiving anti-EGFR antibodies (e.g. cetuximab) for cancer treatment should the need arise. Thus, considering future clinical implications and choosing suitable reporter genes is important. Another way to circumvent this challenge could be to utilize an enrichment strategy relying on transiently expressed reporter genes, thereby allowing treatment with anti-reporter antibodies when expression of the reporter gene is silenced [139]. Integration of two different reporter genes at the *HBB* locus in HSPCs constituted a strategy for enrichment of cells with a biallelic targeted integration in more than 85% of cells. Furthermore, integrating an *HBB* cDNA correcting the sickle cell disease mutation followed by a clinically relevant EF1a-tNGFR cassette for enrichment of anti-sickling HSPCs, confirmed the potential to enrich functionally corrected HSPCs which expressed mRNA from the integrated anti-sickling cDNA driven by the endogenous promoter [140–142]. Similar results were obtained from HDR-mediated integrations at other loci where 99%, 92%, and 100% of reporter-positive HSPCs had at least monoallelic targeted integration at *CCR5*, *IL2RG*, and *RUNX1* respectively [143].

Integrating various cDNAs from genes of interest fused to a fluorescent reporter through an internal ribosome entry site (IRES) has also been demonstrated to be greatly enriched upon selection based on the reporter expression [144]. Also, including an additional reporter gene in the HDR template outside the homology arms further allows for exclusion of cells with random integration events and cells with only episomal HDR template expression by negative selection [145].

Vaidyanathan et al. developed a gene-corrected airway stem cell therapy against Cystic Fibrosis (CF) by targeted replacement of full-length *CFTR* and enrichment by co-integration of a tCD19 reporter. Enrichment of modified cells by immunomagnetic selection obtained 60–80% tCD19 + upper airway basal stem cells (UABCs) and human bronchial epithelial cells (HBECs) from 11 different CF donors. Integration of the full-length *CFTR* cDNA and tCD19 enrichment cassette was confirmed into at least one allele per tCD19+ cell and the corrected airway basal stem cells were able to differentiate to produce epithelial sheets with restored *CFTR*-mediated chloride transport at an average of 70–80% of the levels seen for non-CF controls [60].

So far, the most promising cellular therapies include chimeric antigen receptor (CAR) T cell therapies, which are rapidly emerging as very promising cellular therapies especially for use as cancer immunotherapies, and site-specific gene editing technologies like the CRISPR/Cas system are increasingly used for next-generation engineering and production of CAR T cells [146]. A certain level of site-specific CAR integration is required to meet clinical release criteria to assure proficiency. Therefore, enrichment strategies have also been applied to specifically enrich for the engineering of CAR T cells. Integration of a multi-epitope molecule harboring a CD34 epitope and two CD20 mimotopes (RQR8) along a CD19-targeting CAR into the CD52 locus resulted in 60% of genome-edited T cells being CAR+ /CD20+ /CD34+ /CD52-, which could be increased to > 95% after CD34-based positive selection. A dual functionality of the RQR8 as both a selectable reporter and as a suicide switch sensitive to rituximab (anti-CD20) further advances this type of enrichment strategy for use in CAR T cell engineering [147]. Reporter genes such as tEGFR has also been coupled to CD19-targeting CARs for enrichment of CAR T cells [148]. A more refined approach may be to incorporate a selectable gene fragment within the CAR coding sequence itself. Strep-tag II and *NGFR* sequences have been introduced within the CAR N-terminus, enabling enrichment of CAR T cells to > 90% purity [69, 148].

Abrogating the need for a reporter gene has also been demonstrated by choosing essential genes as integration sites [149]. Here, the reading frame of the essential gene from the location of the target site to the stop codon is included in the repair template to restore the reading frame of the essential gene upon integration. This partial cDNA is followed by the desired transgene to be expressed, and only cells with restored expression of the target gene survives the editing process. Targeting the *GAPDH* locus achieved > 90% transgene integration efficiency into the *GAPDH* locus in primary cells. Furthermore, this strategy also reduces undesired INDELs and incorrect integrations since precise HDR is required for survival of edited cells [149].

### Integration of an excisable reporter

Permanent expression of a selectable reporter gene integrated along a gene of interest to enrich for gene edited cells may interfere with neighboring genes, may be immunogenic, or perturb cell function and homeostasis, which can be especially troublesome in a therapeutic context. To solve this issue, a common strategy is to first integrate a GOI and reporter gene for positive selection of cells with an integration, then subsequently excise the reporter gene using recombinases or transposon systems and a negative selection marker. Only cells with successful removal of the enrichment cassette are included in the final enrichment step. The excision step can be performed by either Flp/FRT or Cre/loxP recombinase systems or an excision-only piggyBac transposon system by surrounding the enrichment cassette with relevant excision target sites [57]. This combined enrichment and excision strategy aims to minimize the impact at the target site since only the intended edit remains.

The Cre/loxP recombinase system can excise fragments flanked by two loxP sites, leaving a single 34 bp loxP “scar” behind [150]. Numerous enrichment strategies have relied on subsequent excision of the enrichment cassette following integration [131, 151–153]. A few studies even demonstrated that expression of the integration transgene increased following excision of the co-integrated enrichment cassette [152, 154].

Since both the Cre/loxP and the Flp/FRT systems leave behind a footprint in the form of a single loxP or FRT site after excision, an alternative method is the piggyBac transposon system which mediates “scarless” excision by removing transgenes flanked by piggyBac-specific inverted terminal repeats (ITRs) if the genetic sequence already contains a simple TTAA site, otherwise this will be left behind [155]. Furthermore, cytotoxicity and genotoxicity caused by prolonged expression of the Cre recombinase is well documented. The same has not been documented for the piggyBac transposase [156, 157]. The piggyBac transposon system has been the preferred choice for excision of enrichment cassettes following enrichment and has been described in numerous studies [122, 145, 157–160].

With the ambition to modify the genome as little as possible, these strategies do require multiple genomic manipulations and enrichment processes which in return can introduce additional risks of cellular- and genotoxicities. PiggyBac excision from heterochromatic regions has also been demonstrated to be far less efficient [157, 161]. Thus, if an integration, enrichment, and excision strategy is performed at a non-expressed genetic locus, then the transposon can be difficult to remove [157]. Another approach could be to excise the enrichment cassette by CRISPR/Cas-mediated excision. However, additive off-target risks and genomic translocations are associated with multiple DSBs. [153, 162]

### Reporter integration with transient expression

As an alternative to enrichment cassette excision following integration, we have recently developed a strategy for enrichment of CRISPR/Cas-mediated transgene integration by transient CRISPR activation (CRISPRa) of an otherwise silent reporter gene. CRISPRa is a fusion complex consisting of catalytically deactivated Cas9 (dCas9) fused to transcriptional activators, for example the tripartite transactivator VP64-p65-Rta (VPR). The CRISPRa complex is directed to the region immediately upstream of the transcriptional start site (TSS) of a target gene by a sgRNA where it activates expression of the target gene [163, 164]. An enrichment cassette consisting of a silent miniCMV promoter driving a therapeutically relevant reporter gene is integrated along a GOI enabling a short transient burst in reporter expression allowing enrichment of cells with targeted integration. Up to 3.6-fold enrichment (from 17.7 to 66.8%) of cells with transgene integration was achieved at various loci (*HBB*, *AAVS1*, *CCR5*) using various reporter genes (*tNGFR*, *tEGFR*, *tCD19*, *tCD8*) in both primary human T cells and HSPCs. Furthermore, enriched CAR T cells displayed improved cytotoxicity over non-enriched cells [139]. This transient enrichment strategy constitutes a novel strategy for enrichment without the risks associated with permanent reporter expression or excision of reporter genes. A similar approach based on transient reporter expression by a Tet-On-driven system instead of CRISPRa has also been developed [165]. However, several Tet-On systems remain compromised by low inducibility and leaky promoter expression. Leaky expression was also observed in our strategy, so additional optimizations may be required for the further use of this type of enrichment strategy [139, 166].

Despite being superior due to the direct enrichment approach, these strategies also face challenges related to the more extensive genome modifications or promoter interference between transgene and reporter promoters. However, all enrichment strategies described in this review are dependent on strong reporter expression for selection to occur. One excision strategy was unable to counter-select for reporter-excised cells after piggyBac transfection, most likely due to silencing of the cassette since a transcriptionally inactive genomic locus was targeted, demonstrating the importance of reporter expression [160]. A comparison of different promoters driving reporter expression did not demonstrate any difference between the percentages of alleles that underwent HDR between any of the different promoter constructs tested, concluding that the promoter choice only affects expression levels and enrichment possibility [142].

## Specialized enrichment strategies

### Enrichment of biallelic editing

Genome modification at low frequencies often occur on only one allele, but for applications such as correction of disease-causing genetic mutations, gene therapies, and development of transgenic cellular or animal models it is highly desired to be able to enrich for cells with biallelic genome modifications. Since biallelic editing efficiencies are even lower than monoallelic editing efficiencies on a per-cell basis, especially for HDR-mediated editing, screening for cells with biallelic editing can be very cumbersome. Instead, specialized enrichment strategies for the enrichment of biallelic genome modifications have been developed (Table 1).

**Table 1:** Biallelic enrichment strategies

Cells	Target locus	Transgenes	Selection	Editing pre-enrichment	Editing post-enrichment	Fold increase	Ref.
HEK293T	APP/PSEN1	Introduction of restriction site/reporter genes	Antibiotic resistance	N.A	< 82%	N.A	[167]
HEK293T	CCR5	Reporter genes	Antibiotic resistance	N.A	34.1%	N.A	[8]
HSPC	ASXL1/RUNX1/ HBB/CCR5	Fluorescent genes	FACS	10.4%	94%	9-fold	[143]
HSPC	ASXL1 and RUNX1	Fluorescent genes	FACS	1.1%	78%	70.9-fold	[143]
iPSC	SNCAe2	Mutation in HA/ Fluorescent genes	FACS	2.1%	100%	46.5-fold	[145]
iPSC	SNCAe3	Mutation in HA/ Fluorescent genes	FACS	3.4%	100%	29.4-fold	[145]
iPSC	PINK1e5	Mutation in HA/ Fluorescent genes	FACS	3.7%	100%	26.6-fold	[145]
iPSC	SERPINA1	Correction of E342K missense point mutation/Reporter genes	Antibiotic resistance	N.A	40%	NA	[160]

*N.A.* not available, *HA* homology arm

**Fig. 3:**
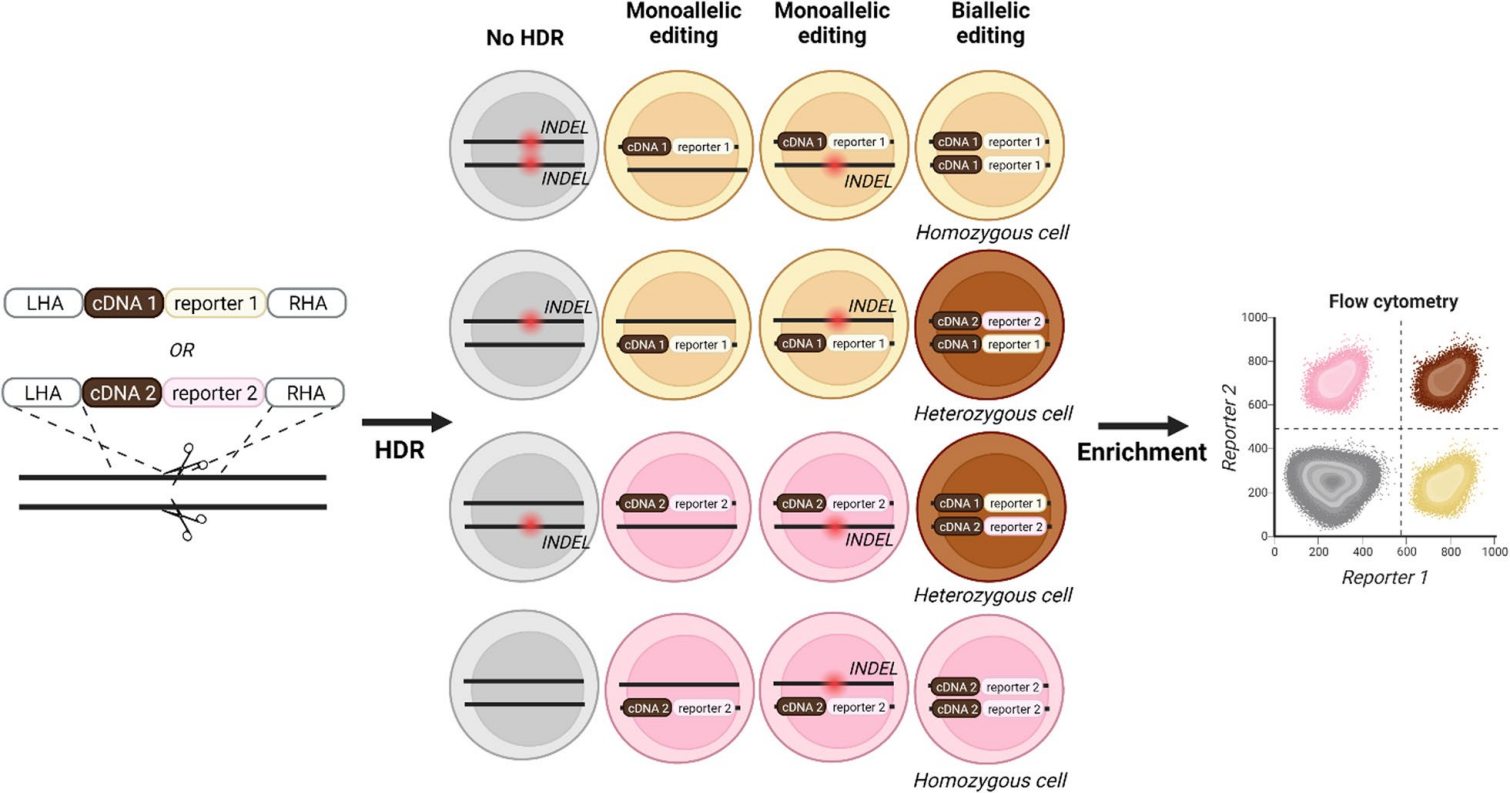
HDR editing outcomes. Constructs with different transgenes, for example specific cDNA variants, can be coupled to different reporter genes to allow for enrichment of different editing events and discrimination between heterozygous and homozygous alleles if different cDNA variants or transgenes are combined. The different possible editing outcomes are depicted. Cells with a desired genotype can be enriched by FACS

A co-targeting approach to impair sensitivity towards Diphtheria Toxin (DT) has been used for enrichment of biallelic integration events at another locus [120]. Sequential targeting of each allele of a gene of interest to directly integrate different reporters allows for enrichment of cells with integrations at both alleles. However, this approach is only able to enrich for cells where different reporter HDR templates are integrated at each allele, thereby overlooking cases where the same HDR template is integrated at both alleles (Fig. 3). Nonetheless, implementing a dual reporter system containing different reporter genes has resulted in highly efficient enrichment of biallelic integration events compared to using only one reporter for enrichment [8, 167]. Enrichment of biallelic editing using two fluorescent reporters were obtained in human iPSCs with either regular CRISPR/Cas HDR-mediated integration [145] or with two opposite-strand nicks by Cas nickase (nCas9) followed by HDR-mediated integration [160], and obtained in human primary T cells and HSPCs as well [143].Furthermore, Bak et al. demonstrated both biallelic, targeted integration of fluorescent reporters for a number of loci (*ASXL1*, *RUNX1*, *HBB*, *CCR5*) as well as di-genic editing, targeted integration at two loci simultaneously (*HBB* and *IL2RG*, *HHB* and *AAVS1*), yielding ~ 10% double positive HSPCs in both cases. Combining both di-genic and biallelic targeted integration, they succeeded in simultaneously targeting both alleles of *ASXL1* or *HBB* and both alleles of *RUNX1* with on-targeted integration at both alleles at both loci in 78% of HSPC clones. This approach allows for a total of six simultaneous genetic modifications: two endogenous genes inactivated (both alleles of each gene) plus the addition of four different transgenes. Furthermore, multi-genic HDR-mediated targeted integration was demonstrated in HSPCs targeting three (*HBB*/*CCR5*/*IL2RG* or *RUNX1*/*HBB*/*ASXL1*) or four (*HBB*/*CCR5*/*ASXL1*/*RUNX1*) loci at once showing successful triple- and quadruple-positive cells with 78% of HSPC clones obtaining the targeted tri-genic integrations at most alleles. These studies demonstrate tracking and enrichment of targeted integration events with both wild type and/or knock out alleles. Integrating a cDNA along the fluorescent reporter thereby allows for widespread applications [143]. This multiplexed HDR approach has been applied to model severe combined immunodeficiency (SCID) with biallelic knockouts of relevant genes and subsequently for gene correction of *RAG2*-SCID patient-derived CD34 + HSPCs by biallelic integration of a complete *RAG2* cDNA. Cells were sorted based on one of the reporters and revealed successful cDNA integration at ~ 50% of all targeted alleles [9]. Other specific heterozygous mutations have also been modeled in HSPCs with this cDNA and fluorescent reporter integration approach [168, 169].

### Enrichment of base editing

As an alternative to standard gene editing approaches that require formation of a DSB, base editors and prime editors, introducing small nucleotide changes independently of HDR, are emerging tools for creating specific genome modifications. The lack of requirement for DSBs and HDR pathway activity results in reduced INDEL rates and potentially higher editing efficiencies in a broader range of cellular contexts. Although base editing efficiencies can be really high, some loci and some cell types can still be challenging to edit, which has led to the development of several strategies for enrichment of base edited cells. Various surrogate reporters to reveal base editing activity has been described based on disruption of a premature stop codon [170–173], resolving a disruptive codon [174], inducing restoration of reporter expression by base excision repair [175], formation of a start codon for reporter expression [171], conversion of one fluorescence to another by a single base edit (BFP to GFP conversion) [176–178], or formation of an endogenous selectable phenotype for co-selection of base editing events [120]. An overview of base editing enrichment strategies is combined in Table 2.

**Table 2:** Base editing enrichment strategies

Strategy	Cells	Target locus	Selection	Editing pre-enrichment	Editing post-enrichment	Fold increase	Ref.
BE-FLARE	PC9	BRAF (T57I)	FACS (BFP to GFP)	9%	55%	6.1-fold	[176]
BE-FLARE	PC9	BRAF (Q58*)	FACS (BFP to GFP)	4%	20%	5-fold	[176]
BE-FLARE	PC9	EGFR (T790)	FACS (BFP to GFP)	1%	18%	18-fold	[176]
TREE	HEK293T	APOE (R158C)	FACS (BFP to GFP)	9%	26%	2.9-fold	[177]
TREE	hPSCs	APOE (R158C)	FACS (BFP to GFP)	6%	23%	3.8-fold	[177]
BIG-TREE	hPSCs	APOE (R158C)	FACS (BFP to GFP)	10%	80–90%	> 8-fold	[178]
GO	DLD1	EMX1/FANCF/CTNNB1	Antibiotic resistance	< 1%	~ 30–60%	10- to 120-fold	[171]
GO	Pancreatic organoid	Intergenic region Chr8/APC	Antibiotic resistance	< 1%	44%	> 44-fold	[171]
Universal toxin-based selection	HEK293T	DPM2/EGFR/EMX1/PCSK9/ DNMT3B	Drug selection	N.A	N.A	4 to 7-fold	[120]
Universal toxin-based selection	HEK293T	EMX1/CTLA4/IL2RA/ AAVS1	Drug selection	N.A	N.A	6 to 13-fold	[120]
Universal toxin-based selection	HCT116	PCSK9	Drug selection	N.A	N.A	13-fold	[120]
ACE	HEK293T	FANCF	FACS	0.2% 15%	58% 78%	290-fold and 5.2-fold	[175]
BEAR	HEK293T	HEK/CCR5/FANCF/SCN5a	FACS	3–10%	20–25%	2.8 to 4.8-fold	[179]
BEAR	HEK293T	HEK/CCR5/FANCF/SCN5a	FACS	5–40%	12–60%	1.3 to 2.9-fold	[179]
BEON	HEK293T	Human site 1 (S1)	FACS	9.5% ± 4.9%	70.6% ± 7.3%	2.4-fold	[172]
BEON	HEK293T	CAR	FACS	N.A	37.54% (± 1.27%)	NA	[172]
BEON	Neuro2A	mTmem5	FACS	N.A	~ 60%	NA	[172]

*N.A.* not available

Several strategies describe a BFP to GFP conversion upon a C-to-T substitution allowing for enrichment of cells with base editing activity. These strategies have shown up to 45-fold increase (in one experiment from ~ 1 to ~ 45%) in the desired base edit and reaching up to 85% editing (in another experiment from 20 to 85% corresponding to a 4.25-fold increase) in primary cells at multiple independent loci with increased multiplexed base editing frequencies as well [176–178].

A system able to enrich for both adenine and cytosine base edits utilizes a split fluorescent gene disrupted by the last intron of the mouse Vim gene that can be restored by correcting a splice donor site by either adenine or cytosine base editors as it allows variable target sequences corresponding to different PAMs and editing windows due to the intronic sequence which can be varied without restrictions. Up to 4.8-fold enrichment and up to 2.9-fold enrichment was achieved on five independent base editing target sites for cytosine and adenine base editors, respectively, based on the less efficient but more precise nuclease deficient Cas9 (dCas9) compared to a nicking Cas9 (nCas9) [179].

Some of the caveats of all base editing approaches are the potential formation of INDELs when using a nicking Cas9 (nCas9) and the potential editing of non-target, bystander bases that are located within the editing window of the sgRNA protospacer. Consequently, this could limit the application of base editing reporters where formation of INDELs or conversion of non-target bases result in mutations disturbing the specificity of the reporter. Thereby, caution should be used when designing these base editing reporters.

### Enrichment of prime editing

Prime editing enables the introduction of short insertions, deletions, and nucleotide substitutions into the genome without requiring a DSB. Prime editors consist of an nCas9 fused to a reverse transcriptase (RT) enzyme. The RT extends the nicked DNA strand using a primer binding site (PBS) and an RNA template embedded in the 3’ terminus of a prime editing sgRNA (pegRNA). pegRNA design is complex since several PBS and RT template combinations are functional in a broad range of cell types and extensive optimization can be required [23]. Despite the great flexibility to modify the genome in almost any possible way, editing efficiency of prime editing is generally low achieving editing rates of around 10–30% [29]. To circumvent these limitations different enrichment approaches have been implemented. An overview of prime editing enrichment strategies is provided in Table 3.

**Table 3:** Prime editing enrichment strategies

Strategy	Cells	Target locus	Selection	Editing pre-enrichment	Editing post-enrichment	Fold increase	Ref.
PEAR	HEK293T	FANCF	FACS	N.A	76%	N.A	[183]
PEAR	K562	FANCF	FACS	N.A	62%	N.A	[183]
PEAR	U2OS	EMX1	FACS	~ 15%	~ 40%	2.67-fold	[183]
PEAR	HUES9	FANCF	FACS		28%	7.8-fold	[183]
PEAR	HEK293T	HEK3/RUNX1/HEXA/ EMX1/DNMT/FANCF/ HBB/PRNP	FACS	N.A	Up to 84%	2.1- to 4.6-fold	[183]
Marker-free co-selection	K562	RNF2/RUNX1/FANCF/ EMX1	Drug selection	N.A	83%	N.A	[184]
Marker-free co-selection	HeLa	RNF2/RUNX1/FANCF/ EMX1	Drug selection	< 1%	26–59%	> 26-fold	[184]
Marker-free co-selection	K562	RUNX1 and FANCF	Drug selection	N.A	81% (RUNX1) and 64% (FANCF)	N.A	[184]
Marker-free co-selection	K562	EMX1 and RUNX1	Drug selection	N.A	63% (EMX1) and 78% (RUNX1)	N.A	[184]
Marker-free co-selection	HeLa	MTOR	Drug selection	4–17%	30–78%	4- to 8-fold	[184]
Marker-free co-selection	U2OS	MTOR	Drug selection	N.A	74–89%	N.A	[184]

*N.A.* not available

Several of the base editing enrichment strategies could be repurposed for enrichment of prime editing events, which in one study was demonstrated to be less efficient as expected but still succeeded in generating reporter positive cells for enrichment [171]. Also strategies based on transfection-positive cells [28, 180] or conversion of fluorescence-based reporters could be used for enrichment of prime edited cells [181, 182].

Simon et al. were the first to develop a prime editor activity reporter (PEAR), relying on a surrogate reporter-based approach to enrich for prime editing based on their previous flexible base editing reporter [179]. The reporter contains an inactive splice site activated by prime editing enabling expression of GFP. Enrichment of nine different endogenous targets with single-nucleotide substitutions, insertions, or deletions achieved co-targeting of the surrogate reporter and an endogenous target editing frequency reaching up to 82% and achieved up to 7.8-fold enrichment of prime editing at the *FANCF* gene [183].

Next, Levesque et al. developed a prime editing enrichment strategy based on co-targeting the endogenous *ATP1A1* gene encoding Na/K ATPase, also repurposed from a previous enrichment strategy. Various loci and modifications were co-selected for in K562 and HeLa S3 cells, and the frequencies of alleles harboring precise modifications markedly increased after enrichment at all tested loci. Furthermore, the enrichment strategy was advanced by identifying multiple *ATP1A1* selectable mutations that allows for sequential enrichment steps by increasing the dose of Ouabain at each step. Successive rounds of enrichment yielded highly modified cells with multiple modifications reaching cells with two different modifications on 88% and 58% of alleles at the *MTOR* locus, respectively [184]. An overview of prime editing enrichment strategies is combined in Table 2.

For both base editing and prime editing, enrichment can alter the purity of the editing product, defined as % intended editing events / % all editing events, as the incidence of unintended edits like INDELs or incorporation of the sgRNA scaffold at the target site will also be enriched. A compromise is to use the less effective dCas9 to avoid nicks resulting in INDEL formation [179, 183, 184]. One base editing enrichment strategy using dCas9 demonstrated up to 30.1-fold increase in product purity compared to using a nCas9 and was able to enrich for base edited cells to achieve the same level of desired modifications as with the dCas9 [179].

### In vivo enrichment

Several of the enrichment reporters described for CAR integrations could also be utilized for depletion of CAR-positive cells in vivo as a safety switch to rapidly remove the immunotherapeutic cells if toxicity is observed [147]. However, only a few studies describe enrichment of gene modified cells in vivo. Selection of gene modified cells in vivo has been reported based on a mutant O6‐methylguanine DNA methyltransferase (MGMT^P140K^) gene that confers resistance to O6‐BG/BCNU (O6‐Benzylguanine/Carmustine) given at doses lower than those used for cancer chemotherapy [185–187]. Efficient selection of genome modified HSCs was demonstrated using both gammaretrovirus and HIV-derived lentivirus vectors in both macaque and baboon nonhuman primate models. Genome modified cells were stable more than 14 months after the last drug treatment, resulting in increased frequencies of transgene-expressing cells from 11.3 to 76.9% for granulocytes, from 15.3 to 49.0% for lymphocytes, from 5.6% to 15.2% for erythrocytes, and from 6.7 to 64.0% for platelets [188]. Recently, MGMT^P140K^-mediated in vivo selection of prime edited cells was reported for correction of the sickle cell disease (SCD) mutation by introducing a T > A conversion. A helper‐dependent adenoviral vector (HDAd5/35++) was used for in vivo delivery of prime editors to target mobilized HSCs in the peripheral blood to correct the SCD mutation in a SCD mouse model (CD46/Townes mice). A MGMT^P140K^ selection cassette was included for selection of transduced cells to confer resistance to O6‐BG/BCNU. Transduced cells were enriched for at days 6, 19, and 33 following vector injection to select for cells with the T > A conversion. The conversion efficiency reached an average of 43.6% at week 16 and was consistently detected in differentiated progeny cells [189]. Enrichment of base editing based on bacterial toxin resistance (DT resistance) was also demonstrated in vivo in a humanized mouse model expressing hHBEGF under the liver cell-specific albumin promoter. The base editing system and sgRNAs for hHBEGF and another endogenous target were delivered using adenoviral vectors (AdV8) and edited cells were enriched by DT treatment two weeks later. This approach resulted in 2.5-fold increase in base editing efficiency at the mouse *Pcsk9* gene compared to control mice [120].

## Discussion and future enrichment strategies

Despite several advancements for genome editing technologies, improvement of quality, safety, and efficiency is still required, particularly for clinical use. Many genome editing technologies are relatively new and their precise mechanisms of action are still being unraveled. While continuously subject to optimization, genome editing frequencies can still be so low that some kind of enrichment strategy is warranted. Enrichment of edited cells might help elucidate and consequently avoid some of the unintended outcomes of genome modification, as improvements to detection methods for gene editing outcomes will also help the engineering of safer and more precise genome modification technologies. It is possible that the combined efforts in improving the gene editing technologies themselves and improving enrichment strategies will aid future enhancements of gene editing efficiencies.

An ideal enrichment strategy would entail a direct selection of precisely edited cells while still being completely “scarless” and only having the desired precise modification at the specified target site. Both unedited cells and cells with unintended edits at on-target or off-target loci should be sorted away. No unintended genomic impact or off-target editing should be introduced, no adverse effects related to the editing or enrichment process should occur, and gentle and scalable selection applicable in all cellular contexts should be possible.

An enrichment strategy able to deselect cells or kill cells with unintended editing outcomes or abnormal DNA damage responses would be advantageous, yet still sought-after. One strategy approaches this by targeted integration into an essential gene, however unedited cells still remains [149]. Other strategies to approach this could include inducible reporter genes that would only be expressed if a specific repair mechanism is active in the cell or a fusion molecule (e.g. dCas9 fusion complex) scanning the genome and inducing cell death if an unintended edit was identified. Development of such strategies would enable implementation of a safety switch in all gene and cellular therapy products allowing for improved safety and control of therapeutic outcomes. Another strategy specifically for in vivo enrichment could be to integrate a unique receptor that signals cell division or cell survival followed by supplying a ligand specific for only that receptor in vivo after editing.

As an alternative to selectable reporter genes for enrichment, smaller structures like RNA aptamers could replace reporter genes to create less of an impact on the cellular genome when integrated due to their small size and simplicity. Fluorescent light‐up aptamers (FLAPs) are RNA sequences that can bind nontoxic cell‐permeable small‐molecule fluorogens thereby aiding visualization and allowing for selection of gene modified cells [190]. The most frequently used fluorogens are derived from 4-hydroxybenzlidene imidazolinone (HBI), the fluorogen moiety in GFP able to bind various FLAPs coupled to mRNA for detection purposes [191]. No enrichment strategy has so far been developed based on FLAPs, which could be due to associated challenges including relatively low brightness, limited thermostability or photostability, scaffold requirement, and detection challenges. However, as FLAPs have been developed in a multitude of colors they pose an interesting approach for enrichment of multiple simultaneous edits if potential challenges can be overcome.

Enrichment of gene modified cells provides a huge potential in basic life science research where engineered cell lines are extensively used. Development and engineering of specific cell lines can in some cases be very inefficient, especially introducing multiple edits for example to study co-dependence of specific proteins can be challenging. In addition, it can be very cumbersome to screen hundreds of clonal cells before finding a clone with the desired edit(s). Enrichment strategies can drastically reduce the number of clones to be screened or completely abrogate it and thereby simplify the engineering process. If the enrichment process is efficient enough, this might enable working with a pool of cells instead of a clone. Several enrichment strategies, spread across all the categories mentioned in this review, have also been specifically developed and used for enrichment of gene edited cells from other organisms and plants [17, 106, 192–195].

Human induced pluripotent stem cells (hiPSCs) are another example of a technology spread across a large number of research areas. iPSCs are used in many research areas including disease modeling, developmental biology, drug screening and development, regenerative medicine, and for generating patient-specific differentiated cells for personalized therapy. Yet, obtaining efficient genetic engineering of iPSCs and maintaining quality is challenging due to infrequent HDR, time-consuming clonal expansion, and low cell viability upon manipulation resulting in generally low editing efficiencies [196]. As a solution, a large number of studies have described enrichment of modified iPSCs [77, 78, 80, 100, 119, 120, 123, 126, 127, 131, 145, 149, 158–160]. Efficient CRISPR/Cas-mediated editing of iPSCs would prove a valuable step towards new therapies against a huge number of diseases and enrichment strategies might help facilitate this goal.

Enrichment of various cell types have already been applied to the clinic. Examples include CD34 enrichment of HSPCs which can boost graft function after allogenic hematopoietic stem cell transplantation and deplete T cells from the donor graft to limit graft versus host disease (GvHD) [197–199], or CD3+ T cell enrichment before viral transduction for CAR T cell therapies [200]. The most common enrichment methods rely on fluorescent activated cell sorting (FACS) or immunomagnetic enrichment / magnetic activated cell sorting (MACS) through direct (primary antibody-conjugated microbeads) or indirect (primary antibody plus secondary antibody-conjugated microbeads) sorting. FACS has the highest level of purity, however suffer from low throughput and yield compared to immunomagnetic enrichment, which in one study resulted in only 7–9% cell loss compared to ~ 70% cell loss for FACS [201]. Furthermore, immunomagnetic enrichment is cheaper and faster for low proportion samples and faster for enrichment of multiple samples due to parallel handling. During FACS, cells are subjected to strong lasers and hydrostatic pressure which might influence cell viability. Nonetheless, cell viability remains high (> 83%) with both methods, however slightly higher for immunomagnetically enriched cells [201]. Devices for clinical grade enrichment in a closed and sterile system have been developed including the CliniMACS Prodigy device (Miltenyi) and the MACSQuant Tyto (Miltenyi) for FACS and immunomagnetic selection, respectively [202], as well as the CellCelector (Sautorius) based on imaging. Both the CliniMACS Prodigy and the MACSQuant Tyto have been extensively used in clinical trials [198, 199, 203–205]. Future enrichment devices might be developed based on levitation of cells between magnets where the position of the cell depends on density, so different cells can be separated using this technique. Similar approaches could be based on cellular weight [206].

Enrichment of gene modified cells have also been used in a number of clinical trials. Neomycin resistance (NeoR) has been used for enrichment of cells transduced with a retroviral vectors, however strong immunogenicity of transduced cells was observed in one trial [197, 207]. Instead tNGFR has been used to magnetically immune-select transduced cells based on tNGFR to enrich for gene modified cells before transplantation into patients in a number of clinical trials [136, 138, 207]. Other clinical studies focusing on the ability to eliminate reactive T cells in the case of graft GvHD have enriched for genetically engineered donor lymphocytes expressing the herpes-simplex thymidine kinase suicide gene fused to CD34 or truncated CD34 (tCD34) before infusion into patients. Enrichment of CD34-TK75- or tCD34-TK75-positive cells yielded an almost pure infusion product and also allowed for tracking of modified cells in the patients. Circulating modified T cells persisted in the patients for over 12 months [205, 208]. Similarly, the use of inducible caspase 9 (iCasp9) as a suicide marker for a safety switch in T cell therapy have been demonstrated by transducing peripheral blood mononuclear cells (PBMCs) with a retroviral vector containing iCasp9 coupled to truncated CD19 (tCD19) through a 2A self-cleaving peptide. The cells were enriched for CD19 by immunomagnetic selection before infusion. iCasp9-transduced T cells were readily detectable 4 weeks post-infusion in all patients and remained at high level (11% of T cells) in one patient alive at 3.6 years [198, 204]. These trials demonstrate the potential to use clinically relevant selection markers for enrichment before infusion and for tracking modified cells post-infusion in patients.

## Conclusion

Enrichment strategies constitute helpful tools to enhance all types of gene modification efficiencies and can be considered in many laboratory and clinical contexts, as they are able to offer high frequencies of genome edited cells, up to 100%, depending on the strategy. Most of the strategies mentioned in this review employ surrogate reporters or selectable transgenes for enrichment, of which the latter option has tried to transition into more clinically appropriate strategies, however not without limitations. Thus, the best enrichment choice depends on the individual situation since each enrichment strategy has both advantages and disadvantages. Nevertheless, enrichment strategies still play an important part in making gene editing technologies applicable, especially when new, exciting, but non-optimized technologies emerge.

## Data Availability

Not applicable.

## Abbreviations

6-TG: 6-Thioguanine
BCNU: N,N′-bis(2-chloroethyl)-N-nitroso-urea (Carmustine)
CAR: Chimeric antigen receptor
Cas: CRISPR-associated
CAST: CRISPR-associated transposon
CF: Cystic fibrosis
CGD: Chronic granulomatous disease
CRISPR: Clustered regularly interspaced short palindromic repeats
CRISPRa: CRISPR activation
CTS: Cas9 target sequence
dCas9: Deficient Cas9
DR: Direct repeats (DRs)
DSB: Double strand break
DT: Diphtheria toxin
FACS: Fluorescent activated cell sorting
FLAP: Fluorescent light‐up aptamer
GOI: Gene of interest
GvHD: Graft versus host disease
HBEC: Human bronchial epithelial cell
HBI: 4-Hydroxybenzlidene imidazolinone
HDR: Homology-directed repair
hiPSC: Human induced pluripotent stem cell
HSC: Hematopoietic stem cell
HSPC: Hematopoietic stem and progenitor cell
HygroR: Hygromycin resistance
iCasp9: Inducible caspase 9
INDEL: Insertion and deletions
iPSC: Induced pluripotent stem cell
iPSC: Induced pluripotent stem cell
IRES: Internal ribosome entry site
ITR: Inverted terminal repeat
MACS: Magnetic activated cell sorting
MGMT: O6‐methylguanine DNA methyltransferase
nCas9: Cas9 nickase
NeoR: Neomycin resistance
NHEJ: Non-homologous end joining
O6‐BG: O6‐benzylguanine
PASTE: Programmable addition via site-specific targeting elements
PBMC: Peripheral blood mononuclear cell
PBS: Primer binding site
PEAR: Prime editor activity reporter
pegRNA: Prime editing sgRNA
PuroR: Puromycin resistance
RT: Reverse transcriptase
SCD: Sickle cell disease
SCID: Severe combined immunodeficiency
sgRNA: Single guide RNA
SSA: Single-strand annealing
TALEN: Transcription activator-like effector nucleases
tCD19: Truncated CD19
tEGFR: Truncated epidermal growth factor receptor
tNGFR: Truncated nerve growth factor receptor
TSS: Transcriptional start site
TwinPE: Twin prime editing
UABC: Upper airway basal stem cell
VPR: VP64-p65-Rta
X-SCID: X-linked severe combined immunodeficiency
ZFN: Zinc finger nuclease
